# Gut Microbiome, Inflammation, and Cerebrovascular Function: Link Between Obesity and Cognition

**DOI:** 10.3389/fnins.2021.761456

**Published:** 2021-12-06

**Authors:** Lisette Olsthoorn, Debby Vreeken, Amanda J. Kiliaan

**Affiliations:** ^1^Department of Medical Imaging, Anatomy, Radboud University Medical Center, Donders Institute for Brain, Cognition, and Behavior, Nijmegen, Netherlands; ^2^Department of Bariatric Surgery, Vitalys, Rijnstate Hospital, Arnhem, Netherlands

**Keywords:** obesity, gut microbiome, inflammation, cerebrovascular function, cognition, brain structure and function

## Abstract

Obesity affects 13% of the adult population worldwide and this number is only expected to increase. Obesity is known to have a negative impact on cardiovascular and metabolic health, but it also impacts brain structure and function; it is associated with both gray and white matter integrity loss, as well as decreased cognitive function, including the domains of executive function, memory, inhibition, and language. Especially midlife obesity is associated with both cognitive impairment and an increased risk of developing dementia at later age. However, underlying mechanisms are not yet fully revealed. Here, we review recent literature (published between 2010 and March 2021) and discuss the effects of obesity on brain structure and cognition, with a main focus on the contributions of the gut microbiome, white adipose tissue (WAT), inflammation, and cerebrovascular function. Obesity-associated changes in gut microbiota composition may cause increased gut permeability and inflammation, therewith affecting cognitive function. Moreover, excess of WAT in obesity produces pro-inflammatory adipokines, leading to a low grade systemic peripheral inflammation, which is associated with decreased cognition. The blood-brain barrier also shows increased permeability, allowing among others, peripheral pro-inflammatory markers to access the brain, leading to neuroinflammation, especially in the hypothalamus, hippocampus and amygdala. Altogether, the interaction between the gut microbiota, WAT inflammation, and cerebrovascular integrity plays a significant role in the link between obesity and cognition. Future research should focus more on the interplay between gut microbiota, WAT, inflammation and cerebrovascular function to obtain a better understanding about the complex link between obesity and cognitive function in order to develop preventatives and personalized treatments.

## Introduction

Worldwide obesity had nearly tripled since 1975 (WHO, 2017), and the prevalence of obesity is still growing (Janssen et al., 2020). Obesity is defined as a condition of excessive fat accumulation, resulting in a body mass index (BMI) of 30 kg/m^2^ or more (Table 1; Engin, 2017). Especially midlife obesity is associated with diabetes mellitus type 2, hypertension and other cardiovascular diseases (Arnoldussen et al., 2014; Miller and Spencer, 2014). Moreover, obesity is related to structural changes in the brain, such as reduced total gray matter (GM) and white matter (WM) volumes (Karlsson et al., 2013; Zhang et al., 2018; Hamer and Batty, 2019). Thus, obesity results in an increased risk of cognitive impairment and dementia in later life (Kivipelto et al., 2005; Chuang et al., 2016; Yang et al., 2018). However, mechanisms underlying these pathologies are still unclear. Research suggests contributions of various factors, such as inflammation, the gut microbiome, and cerebrovascular factors. For example, expanding adipocytes in white adipose tissue (WAT) and especially visceral adipose tissue (VAT), lead to activation of immune cells and the production of pro-inflammatory cytokines in especially VAT, resulting in both local and systemic low-grade inflammation (Hajer et al., 2008; Gnacinska et al., 2009; Illan-Gomez et al., 2012; Crispino et al., 2020). This affects both peripheral and cerebral vascular health and consequently impairs cerebral blood flow (CBF) (Sweeney et al., 2018; Youwakim and Girouard, 2021). Moreover, obesity is associated with changes in the composition of gut microbiota, where certain gut microbiota compositions are associated with increased gut permeability, leading to pathogen infiltration and increased inflammation. These processes are interconnected, and how they may affect cognitive function in obesity, is not yet clear. Of course, many other factors play a role in the link between obesity and cognitive function, such as physical activity, dietary patterns and genetics; however, this is beyond the scope of this review. Further understanding of all these processes and their interplay is important to develop and improve existing treatments, especially since obesity is associated with neurodegenerative diseases, such as Alzheimer’s disease.

**TABLE 1:** Classification of underweight, healthy, overweight, and obesity categories using BMI.

Classification	BMI (kg/m^2^)
Underweight	<18.5 kg/m^2^
Healthy	18.5–24.9 kg/m^2^
Overweight	25.0–29.9 kg/m^2^
Obesity	≥30.0 kg/m^2^

*BMI, Body Mass Index. Adapted from WHO (2017).*

In the present review, we will discuss the current research of the past decade regarding cognitive decline in obesity. Specifically, we will first discuss observed changes in brain structure and cognition in obesity, and then look at contributions of the gut microbiota, WAT, inflammation, and cerebrovascular factors, in both preclinical and clinical studies. For this purpose, recent literature, published in English between 2010 and March 2021 was collected, using PubMed and WebofScience. The following search terms were used in different combinations: “obesity,” “brain,” “function,” “structure,” “cognition,” “inflammation,” “gut,” “microbiome,” “gut-brain axis,” “blood brain barrier,” “neurovascular function,” “cerebral blood flow,” and “WAT.” Furthermore, we included relevant additional publications identified from bibliographies from retrieved literature.

## Brain Structure in Obesity

Obesity is associated with GM and WM volume alterations in the brain. Moreover, increases in waist-to-hip ratio (WHR) during midlife (mean age of 54 years) were associated with decreasing total brain volume 10 years later (Debette et al., 2011). Furthermore, obesity is positively associated with frontal and temporal cerebral cortices thinning (Shaw et al., 2017; Franz et al., 2019), and increasing BMI especially in later life is further positively associated with cortical thinning in the posterior cingulate, right lingual gyrus, anterior cingulate, and peri-calcarine sulcus (Shaw et al., 2017). Adults experiencing obesity show lower hippocampal (Ambikairajah et al., 2020; Hung et al., 2020) and hypothalamic volumes compared to adults with a BMI below 25 kg/m^2^ (Puig et al., 2015). Additionally, increased BMI and waist circumference (WC) are associated with decreased WM volume (Driscoll et al., 2012; van Bloemendaal et al., 2016). Other obesity measures also show associations with brain volumes: for example, body fat percentage is positively correlated with left medial orbital frontal cortex volume and cerebellar WM (Kakoschke et al., 2019). However, multiple obesity measures show different associations and correlations with brain volumes; Ronan et al. (2016), for example, even found no interaction or effect of BMI on cortical surface area, cortical thickness, or cognitive performance.

Next to volumetry alterations, obesity is also associated with an affected microstructure of the brain, such as WM integrity loss (van Bloemendaal et al., 2016), higher diffusivity and lower axonal density (Samara et al., 2020). Indeed, increasing BMI and WHR are positively associated with lower fractional anisotropy (FA) in WM tracts (Stanek et al., 2011; Verstynen et al., 2012; Zhang et al., 2018), and BMI is associated with higher diffusion in frontal and temporal WM, which also correlates with decreased executive functioning and memory performance in older adults (Ryan and Walther, 2014). Increased VAT is further associated with lower FA, while interestingly, higher total fat mass is associated with higher FA (Cardenas et al., 2020).

Obesity is associated with WM loss as represented in white matter hyperintensities (WMH). WMH are markers of cerebral small vessel disease, a disorder of the arterioles and capillaries in the brain linked to decreased cognitive function (Wardlaw et al., 2019). Multiple visceral obesity markers, such as WHR, are positively associated with the presence of deep WMH, and both WC and VAT were shown to predict WMH volume (Pasha et al., 2017; Lampe et al., 2019). Specifically, adults with obesity showed WMH in the left mediobasal hypothalamus, which was further positively associated with increased hypothalamic inflammation (Kreutzer et al., 2017). In a longitudinal study in patients with symptoms of cerebral small vessel disease, obesity was associated with increased WMH volumes later in life (Arnoldussen et al., 2019).

Overall, obesity shows a negative impact on the brain, including reduced brain volumes, WM and GM integrity loss. However, different results between obesity indexes should be considered carefully within the relationship between obesity and brain structure.

## Obesity and Cognition

Multiple human studies focused on the association between obesity and cognitive performance. Cognitive performance has been measured in multiple larger cohorts in human adults in comparison to various obesity indices [e.g., the Brain Resource International Database (BRID) cohort (Gordon, 2003), the Whitehall II Study (Singh-Manoux et al., 2018), the Baltimore Longitudinal Study of aging (Gunstad et al., 2010), the Gothenburg H70 Birth cohort (Sterner et al., 2019), and the Longitudinal Assessment of Bariatric Surgery (LABS) cohort (Gunstad et al., 2011)]. For example, BMI was associated with decreased attention, processing speed, motor function and executive function, but not with language or verbal memory (Stanek et al., 2013; Kesse-Guyot et al., 2015). In a cross-sectional UK Biobank study, scores on fluid intelligence and short term memory were inversely associated with the probability of being overweight and obese rather than being lean. Executive function was only inversely related to the probability of being obese compared to being lean (Olivo et al., 2019).

In contrast to BMI, which is often considered as a measure of total body adiposity, WC and WHR are rather a measure of abdominal obesity, and indeed show different associations with cognition compared to BMI. For example, WC was inversely associated with the rate of cognitive decline while BMI was not significantly associated with cognitive decline in mid- and late life African Americans (West et al., 2017). WHR was furthermore shown to predict episodic memory performance 10 years later whereas BMI was not associated with any cognitive measure (Hartanto et al., 2019). In another study, WHR was associated with decline in processing speed and executive function, though not with memory (Gardener et al., 2020). Moreover, these associations did not hold when controlling for insulin resistance and diabetes (Gardener et al., 2020). The analysis, however, did show that WHR had a stronger association with cognitive decline in adults experiencing obesity compared to lean or overweight adults (Gardener et al., 2020). Thus, multiple studies indicated that WHR and WC may be more sensitive indices in relation to cognitive function in obesity compared to BMI alone. BMI only includes body weight and height, and does not discriminate between weight of muscles and fat, nor the distribution of fat across the body, which differs across age, gender, and race, whereas WC only includes the circumference of the waist (Kadowaki et al., 2018). WHR gives more information about the distribution of fat across the body, but still does not give the complete picture. Direct imaging of WAT and muscle mass showed that while lean muscle mass was associated with better fluid intelligence, non-visceral WAT was associated with worse fluid intelligence (Klinedinst et al., 2019). Non-visceral WAT and VAT have different metabolic effects, which might explain the different effects of obesity indices on cognition (Kiliaan et al., 2014). More information about different WAT depots and fat distribution in relation to cognition is discussed later.

Moreover, BMI and WHR seem to have a different effect on cognition during midlife compared to later in life. Whereas higher BMI at midlife is positively associated with cognitive decline and increased risk of dementia, the opposite is often described in later life (Kiliaan et al., 2014; Pedditzi et al., 2016). In contrast, in a study on aging, obesity at midlife was not associated with cognitive decline later in life (Deckers et al., 2017). WC showed stronger associations with cognition and cognitive decline compared to BMI, although most associations did not remain significant after controlling for age since individuals with obesity tended to be older in their study population (Deckers et al., 2017). Other obesity associated measures show inverse associations with cognition as well; hypertension and systolic blood pressure were for example associated with a decline in executive function (Debette et al., 2011).

More evidence about the link between cognition and obesity measures derives from weight loss studies, as previous studies have observed cognitive improvement after weight loss that followed bariatric surgery (Gunstad et al., 2011; Alosco et al., 2014). Overall, there is a consistent link found in literature between obesity indices and cognitive function, however, the exact mechanisms are still unsolved.

## Gut Microbiome in Obesity

One of the proposed mechanisms involved between obesity and cognitive function concerns the gut microbiome. As dietary patterns largely influence the gut microbiome, it may not be surprising that a high fat diet (HFD) in animals affects gut health and its microbiome. Looking at gut microbiota composition, intake of HFD in mice and rats is associated with decreased microbiota diversity, increased *Firmicutes* and *Oscillibacter* abundance, less *Bacteroidetes* and *Lactobacillus* and increased *Firmicutes*/*Bacteroidetes* ratio (Lam et al., 2012; Hamilton et al., 2015). These changes in abundance were closely associated with increased body weight in mice (Lam et al., 2012). In contrast, mice that showed resistance to diet-induced obesity (DIO) through HFD had a shift toward a lean microbiota composition phenotype compared to DIO mice. Interestingly, these mice also showed lower levels of inflammation and no impairment in memory, whereas the DIO mice showed impaired spatial recognition and discrimination (Zhang et al., 2019).

**FIGURE 1:**
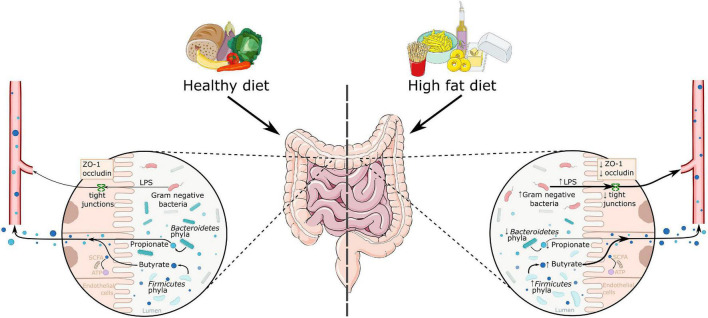
A schematic overview of the effects of a high fat diet on the gut microbiome. High fat diet is associated with increased *Firmicutes* phyla and decreased *Bacteroidetes* phyla abundance. This leads to a change in the produced metabolites, as there is an increase in butyrate and a decrease in propionate levels. High fat diets are also associated with increased Gram-negative bacteria, which activate the immune system via LPS. This LPS can enter the bloodstream through decreased gut permeability, as seen in decreased levels of tight junction proteins zonulin-1 and occludin. ATP, adenosine triphosphate; LPS, lipopolysaccharide; SCFA, short chain fatty acids; ZO-1, zonulin-1.

The gut microbiota is involved in energy metabolism, partly through fermenting complex dietary fibers into short-chain fatty acids (SCFAs) (Baothman et al., 2016). The main SCFAs present in the gut are acetate, propionate, and butyrate, of which acetate and propionate are mainly produced by *Bacteroidetes*, and butyrate by *Firmicutes* (see Figure 1; Baothman et al., 2016). SCFAs are highly important for gut health by maintaining the mucosal epithelium and are used as energy source for gut epithelial cells (Davis, 2016; Xu et al., 2018). Some studies showed that SCFAs increase tight junction proteins in the gut therewith increasing gut barrier integrity (Stilling et al., 2016). A transgenic mouse model for obesity showed impaired gut permeability in the colon (Schroeder et al., 2020). Moreover, HFD induced obesity in mice decreased tight junction protein zonulin-1 (ZO-1) causing increased gut permeability (Lam et al., 2012, 2015). Gut permeability is also affected by the microbiome (Leigh and Morris, 2020), as for example an increased amount of *Oscillibacter* is associated with decreased expression of ZO-1 (Lam et al., 2012). *Oscillibacter* is a Gram-negative species, which contains lipopolysaccharide (LPS). LPS may change the structure of tight junction proteins, thereby increasing gut permeability (Muscogiuri et al., 2019). Thus, many animal studies show higher levels of LPS and increased permeability in the gut in obesity, which may contribute to increased systemic inflammation.

As mentioned above, some studies showed that SCFAs increase the amount of tight junction proteins in the gut thereby increasing gut barrier function (Stilling et al., 2016). For example, obese and diabetic db/db mice showed lower occludin and ZO-1 expression in the colon (Cheng et al., 2018), whereas supplementation of sodium butyrate increased ZO-1 expression in the gut (Xu et al., 2018). A high fiber diet has also been shown to increase SCFA production in the gut and was shown to attenuate inflammatory cell infiltration in mice (Matt et al., 2018). Furthermore, butyrate, when administered with HFD in mice, is found to be protective against DIO (Lin et al., 2012) and neuroinflammation (Arnoldussen et al., 2017). However, *ex vivo* studies in human biopsies of the colon showed that butyrate did not increase tight junction proteins, such as occludin (Tabat et al., 2020). Therefore, these discrepancies between animal and human studies (*ex-vivo*) need further research.

Interestingly, these obesity-associated changes in gut microbiota composition have been shown to be reversable. When ob/ob mice received microbiota from lean mice on a standard chow diet, their microbial composition shifted to a more healthy microbial composition. This was associated with increased SCFA levels while gut permeability and body weight continued to be higher in obese mice compared to the lean mice (Battson et al., 2019). Vice versa, transplanting microbiota from obese mice to lean mice induced increased inflammation and gut permeability (Bruce-Keller et al., 2015). Administering *Akkermansia muciniphila* to HFD-induced obese mice reduced body weight and fat mass (Everard et al., 2013; Plovier et al., 2017), reduced gut permeability (Plovier et al., 2017; Ashrafian et al., 2019; Ou et al., 2020), and reduced inflammation in mice (Plovier et al., 2017). These results show both the effect of diet on microbiota, as well as the impact of microbiota on body weight and inflammation.

Gut microbiota and gut permeability are likewise associated with peripheral and central inflammation. Db/db mice showed increased levels of pro-inflammatory interleukin 1β (IL-1β), both in the hypothalamus and in the intestine (Duparc et al., 2011). In mice, HFD-induced obesity increased macrophage infiltration and expression of pro-inflammatory cytokines such as tumor necrosis factor α (TNF-α) and interleukin 6 (IL-6) in mesenteric WAT, and increased TNF-α expression in the gut (Lam et al., 2012, 2015). Another study showed that HFD-induced obesity in mice increased the proinflammatory M1 macrophage phenotype levels in the colon (Zhang et al., 2019).

One mechanism involved in the association between gut microbiota and inflammation may be the increase in Gram negative bacteria in obesity (such as *Bacteroidetes* and *Oscillibacter*), which contain LPS (de La Serre et al., 2010; Muscogiuri et al., 2019). As mentioned earlier, LPS may change the structure of tight junction proteins, through which it may increase gut permeability (Muscogiuri et al., 2019). Thereby LPS can cross the gut barrier into the circulation, bind to Toll-like receptor 4 (TLR4) and lead to inflammation by stimulating pro-inflammatory cytokine production and activating the innate immune system (Saad et al., 2016). However, the causality of these associations is still unknown.

In humans, adults with obesity showed increased gastroduodenal permeability (though not in the ileum or colon) and a distinct microbiome composition compared to lean adults, with lower microbial diversity and an increased ratio of *Firmicutes*/*Bacteroidetes* (Verdam et al., 2013). The distinct gut microbial composition in adults with obesity was associated with inflammation, as reflected in increased high-sensitivity C-reactive protein (CRP) plasma levels (Verdam et al., 2013). Another study demonstrated that a healthy eating pattern in humans (which included more fruit, yogurt, and less sugar) compared to a less healthy diet showed improvements in both microbiota diversity and inflammation, in the blood as well as in WAT (Kong et al., 2014). In WAT, lower levels of circulating pro-inflammatory monocyte chemoattractant protein-1 (MCP1) and a shift toward anti-inflammatory M2 macrophages were seen (Kong et al., 2014).

Supplementary Table 1 gives an overview of the studies discussed and Figure 1 provides an illustrative summary focusing on the effects of a HFD on the gut microbiome.

### Gut Microbiome and Cognition

Moreover, the gut microbiome is associated with changes in cognition and brain structure. For example, germ-free mice exhibit memory impairment (Gareau et al., 2011), and an antibiotics treatment in mice is associated with reduced object recognition (Frohlich et al., 2016). Hence, gut microbiota may affect cognition in mice. HFD feeding in rats showed an increase in *Firmicutes/Bacteroidetes* ratio and increased serum LPS after a few weeks, as well as reduced learning after 12 weeks (Saiyasit et al., 2020b). Interestingly, HFD-induced obese mice show a reduced occludin and ZO-1 expression in the gut, as well as increased gut inflammation, which was accompanied by impaired spatial and object recognition memory. Object recognition memory was furthermore positively associated with *Bacteroidetes* abundance. DIO resistant mice showed no increased gut permeability, gut inflammation, or memory impairment (Zhang et al., 2019). Interestingly, when mice on a standard chow diet received microbiota from HFD-induced obese mice, they showed increased inflammation, gut permeability, blood-brain barrier (BBB) permeability, anxiety and decreased memory performance (Bruce-Keller et al., 2015). Furthermore, mice that received microbiota from humans experiencing obesity, showed decreased inhibition, just like the human donors, compared to lean human donors and mice who received their microbiota (Arnoriaga-Rodriguez et al., 2021). Altogether, this indicates the importance of a healthy gut microbiota for optimal cognitive function in animals.

**FIGURE 2:**
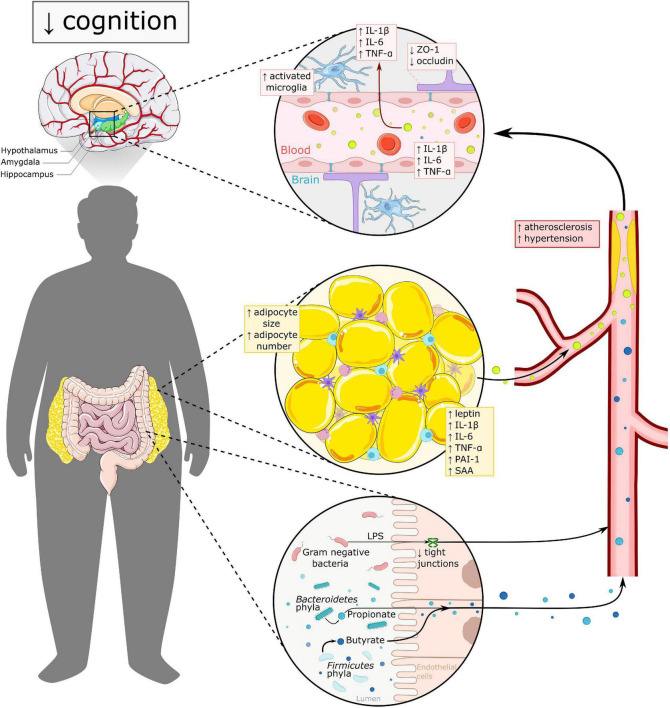
Overview of mechanisms underlying cognitive impairment in obesity. Both excess WAT and altered gut microbiota have a direct and indirect effect on brain functioning. In dysbalanced WAT, adipocytes secrete pro-inflammatory adipokines in the circulation, leading to a more pro-inflammatory state. Adipokines like leptin, PAI-1 and SAA thereby, affect vascular health via promoting atherosclerosis, hypertension and thrombosis. Gut microbiota in obesity is linked to higher levels of LPS and increased gut permeability, which may contribute to increased systemic inflammation. Both excess WAT and altered gut microbiota add to blood brain barrier (BBB) dysfunction, which leads to increased neuroinflammation amongst other. The hypothalamus, amygdala and hippocampus seem to be the most vulnerable regions for obesity related changes and are all three highly important in cognitive functioning. IL-1β, interleukin 1β; IL-6, interleukin 6; PAI-1, plasminogen activator inhibitor 1; TNF-α, tumor necrosis factor α; SAA, serum amyloid; ZO-1, zonulin-1; LPS, lipopolysaccharide.

In humans, studies have also shown associations between gut microbiota and cognition. *Firmicutes* bacteria are positively associated with memory performance, whereas *Bacteroidetes* and *Proteobacteria* are inversely associated with memory (Arnoriaga-Rodriguez et al., 2020). Adults with obesity showed lower scores in a Stroop test, which was positively associated with *Eubacterium* and *Firmicutes* bacterium abundance, and inversely associated with *Bacteroidetes* abundance (Arnoriaga-Rodriguez et al., 2021). One way the gut microbiota may influence cognition is through inflammation: Kreutzer et al. (2017) found increased inflammation in the mediobasal hypothalamus in adults with obesity, which was inversely correlated to *Parasutterella* sp. (*Proteobacteria*) and *Marinilabiliaceae* (*Bacteroidetes*). Furthermore, when mice received fecal transplants from these humans experiencing obesity, they showed decreased memory function, as well as increased inflammatory gene expression in the prefrontal cortex (Arnoriaga-Rodriguez et al., 2020). Thus, research indicates that gut microbiota is one of the factors which may affect cognition through inflammation (see Figure 2).

Supplementary Table 2 gives an overview of the studies discussed based on the influence of gut microbiota on cognition.

## Adipokines, Inflammation, and Cognition in Obesity

### White Adipose Tissue Inflammation and Adipokines

Obesity has been associated with increased peripheral inflammation as shown in multiple human studies. This inflammation is described to start in excess WAT, where adipocytes increase in size as they store more free fatty acids (FFA). In the context of obesity, the adipocytes may also produce more FFA, attracting pro-inflammatory cells, such as macrophages and mast cells to the adipose tissue. Macrophages subsequently shift to a proinflammatory M1 phenotype, and the adipocytes secrete pro-inflammatory adipokines such as leptin, TNF-α, IL-1β, and IL-6 (Illan-Gomez et al., 2012; Castanon et al., 2014; Asghar and Sheikh, 2017), thereby inducing further activation of the immune system. Adipokines have several functions among others involvement in thrombosis, hypertension, metabolism and inflammation. For example, leptin regulates metabolism and contributes to vascular disease via atherosclerosis and thrombosis (Wang et al., 2014) and plasminogen activator inhibitor-1 (PAI-1) is involved in inhibiting fibrinolysis and forming atherosclerotic plaques and hypertension (Kiliaan et al., 2014; Bilgic Gazioglu et al., 2015). Another inflammatory adipokine which shows increased levels in obesity is serum amyloid A (SAA). SAA is considered a biomarker for inflammation as well as for cardiovascular disease, indicating the strong inverse correlation between inflammation and vascular health (Zhao et al., 2010; Deguchi et al., 2010). Altogether, excess WAT in obesity is associated with decreased fibrinolysis, which increases the risk of thrombosis and atherosclerosis, which in the end affects vascular health as well (Figure 2; Kiliaan et al., 2014).

Moreover, by releasing cytokines into the circulation, a low but chronic systemic inflammation is induced (Illan-Gomez et al., 2012). For example, bariatric surgery patients with higher levels of circulating LPS show increased inflammation in VAT and subcutaneous adipose tissue (SCAT), including infiltration of macrophages and increased levels of IL-6 and MCP1 (Clemente-Postigo et al., 2019). However, inflammatory markers have been shown to differ between SCAT and VAT with a more proinflammatory profile in VAT (McLaughlin et al., 2014).

The reviewed studies focusing on WAT inflammation, adipokines in obesity are summarized in Supplementary Table 3.

### Neuroinflammation

Obesity-associated inflammation is not limited to the peripheral parts of the body, but it is observed in the brain as well. Neuroinflammation is observed in neurodegenerative diseases and is seen as a mediator of cognitive impairment (Kumar, 2018). Many animal studies have shown the neuroinflammatory effects of HFD mainly on the amygdala, hippocampus, and hypothalamus, structures involved in regulating emotional behavior, learning and memory, and homeostasis, respectively (Guillemot-Legris and Muccioli, 2017).

Both HFD-induced obesity and transgenic mice models of obesity show inflammation in the hypothalamus (Buckman et al., 2013). DIO in mice showed increased expression of TNF-α and IL-6 in the hypothalamus, and increased TNF-α only in male mice (Lainez et al., 2018; Park et al., 2019). Furthermore, it has been shown that in HFD-induced obese rodents cellular immune responses are activated as seen as increased microglia and astrocyte activation in the hypothalamus (Thaler et al., 2012; Baufeld et al., 2016; Lee et al., 2018). A db/db mice model showed increased inflammation (IL-1β and TNF-α) in the hypothalamus (Duparc et al., 2011), as well as in VAT and the hippocampus (Erion et al., 2014). Interestingly, this increased inflammation was associated with alterations in cognitive function and could be transferred to wild type mice by WAT transplantation or attenuated by exercise and IL-1β receptor blocker via IL-1 receptor antagonist infusion in the hippocampus (Erion et al., 2014). This thereby highlights both the role of VAT as a source of inflammation affecting other organs in the body, as well as the role of IL-1β and its receptors in neuroinflammation.

In mice, HFD intake for approximately 4 months (exactly 16 and 18 weeks) is associated with increased IL-1β and TNF-α expression in the amygdala and hippocampus, which correlated with cognitive impairment and specifically decreased spatial learning and memory performance (Almeida-Suhett et al., 2017; Gladding et al., 2018). Furthermore, HFD induced obese rats showed increased oxidative stress and activated microglia in the hippocampus as well as decreased dendritic spine density (Saiyasit et al., 2020a). Ob/ob mice showed also inflammation, BBB leakage and oxidative stress in the hippocampus (Jin et al., 2020). While HFD for 3 months additionally showed increased synaptic internalization by microglia in the hippocampus, as well as impaired memory, which could be reversed by switching the mice to a low-fat diet for 2 months (Hao et al., 2016), highlighting both the effect of diet, as well as the plasticity of the brain.

In human post-mortem brain tissue, a BMI above 30 kg/m^2^ was correlated with an increased amount of activated microglia in the hypothalamus (Baufeld et al., 2016), as well as with lower anti-inflammatory IL-10 and higher inducible nitric oxide synthase mRNA expression levels in the frontal cortex, which also showed thinner cortices in obesity (Lauridsen et al., 2017; Shaw et al., 2017; Franz et al., 2019). Furthermore, peripheral inflammation markers are inversely associated with cognition. Higher lean muscle mass and lower non-VAT and VAT mass were associated with better fluid intelligence in older adults, and was mediated by circulating leukocytes (Klinedinst et al., 2019). Serum inflammation markers are also associated with structural changes in the brain. For example, metabolic risk factors, including the presence of diabetes, hypertension and obesity, were associated with a thinner cortical thickness of the inferior frontal gyrus, and was mediated by higher serum pro-inflammatory interleukin 2 levels (Kaur et al., 2016).

A recent method used to indirectly measure neuroinflammation is imaging water content in the brain. Here, a higher free water content is hypothesized to indicate increased neuroinflammation. Indeed, results mirror those found in animal models as BMI was associated with higher free water content, mainly in the cerebellum, subcortical areas, and the WM tracts between these areas (Kullmann et al., 2020), as well as in WM of the hypothalamus, hippocampus, and amygdala of adults with obesity, compared to lean adults (Puig et al., 2015; Samara et al., 2020). Further, in individuals with the highest inflammation values, associations were found between higher neuroinflammation and higher BMI, fat mass, CRP, and worse overall cognitive performance (Puig et al., 2015).

The reviewed studies focusing on neuroinflammation are summarized in Supplementary Table 4.

## Cerebrovascular Function in Obesity

Obesity and especially increased VAT is highly associated with hypertension (Hall et al., 2015; Le Jemtel et al., 2018) and atherosclerosis (Alexopoulos et al., 2014; Csige et al., 2018). Adipokines can directly regulate this link between obesity and vascular function via their influence on endothelial cells, arterial smooth muscle cells and macrophages in the vessel wall (Ntaios et al., 2013). Nowadays, vascular health is thought to be an important mediator in the link between obesity and cognitive function.

Moreover, multiple studies showed associations between co-morbidities of obesity such as hypertension and atherosclerosis and cognitive decline. Underlying mechanisms involved in development of cognitive dysfunction and linked to hypertension may include, among others, cerebral vessel remodeling, endothelial dysfunction and oxidative stress (Mansukhani et al., 2019). Furthermore, atherosclerosis is characterized by elevated low-density lipoproteins, that become oxidized which subsequently attracts macrophages, in the end leading to a chronic state of inflammation (Cunningham and Hennessy, 2015). As hypertension and atherosclerosis often co-occur in individuals with obesity, the link between obesity and cognitive function is often mediated by these obesity-related comorbidities (Dye et al., 2017).

Cognitive impairment in obesity is hypothesized to be associated with impaired cerebrovascular function. Mild obese mice showed less glucose transporter 1 (GLUT-1) in the endothelium of blood vessels in the hippocampus and thalamus compared to control mice (Arnoldussen et al., 2016). GLUT-1 is important for glucose uptake from the blood into in the brain tissue. Less GLUT-1 in these areas, however, did not show differences in behavioral tests measuring cognitive function (Arnoldussen et al., 2016). Another study using a high fat, high sugar diet (HFHS) to induce obesity in rats also showed reduced GLUT-1 expression in the hippocampus, as well as learning impairment compared to rats on a control diet (Hargrave et al., 2015).

HFD-induced obesity in mice is furthermore associated with increased vasodilation in cerebral vessels, as well as a higher vascular density in the brain (Cao et al., 2019). In the brain of obese Zucker rats, vasodilation, a decreased inner diameter of the middle cerebral artery and decreased nitric oxide bioavailability was observed (Katakam et al., 2012; Brooks et al., 2015). In Wistar rats, 8 weeks of HFD caused cerebrovascular dysfunction and reduced CBF (Li et al., 2013). Overall, impaired neurovascular function, especially during midlife, is associated with impaired cognition and increased risk of dementia (Iadecola and Gottesman, 2019).

In humans, adults with obesity showed significantly reduced cerebrovascular reactivity (CVR) compared to lean adults. Weight, BMI, and WC were furthermore inversely associated with CVR in a simple linear model in the complete study population (adults with and without obesity) (Rodriguez-Flores et al., 2014). However, in another study when the entire study group was considered (lean controls and adults with overweight or obesity), this inverse association between BMI and CVR disappeared after controlling for insulin resistance (Frosch et al., 2017). Compared to lean adults, adults experiencing obesity showed decreased overall CBF in the brain when performing a response inhibition and attention test (Willeumier et al., 2011), whereas women with obesity showed decreased CBF in sensorimotor areas compared to lean women, and actually showed increased CBF in areas related to the salience network and default mode network when at rest (Silvah et al., 2020). This increased CBF was positively associated with body fat percentage assessed by bio-electrical impedance analysis (Silvah et al., 2020). Furthermore, decreased regional CBF in the left frontal superior orbital and right frontal cortex, cerebellum, right precentral and right postcentral cortex was correlated with increasing BMI (Willeumier et al., 2011). Tarantini et al. (2015) found impaired learning and memory when pharmacologically inducing neurovascular uncoupling in mice which also illustrates this impaired mechanism. However, they did not find changes in synaptic function of neurons in the hippocampus, and underlying mechanisms are therefore not yet clear (Tarantini et al., 2015).

Obesity-associated changes in inflammation and vascular function might lead to increased BBB permeability. It has been shown using radioactively labeled triolein that triglycerides can cross the BBB and affect leptin sensitivity in the brain, specifically in the striatum, hypothalamus, occipital cortex, cerebellum and the midbrain (Banks et al., 2018). BBB leakiness in mice was associated with hippocampal inflammation as demonstrated with increased cytokine production, macrophage infiltration, and cognitive impairment (Stranahan et al., 2016). Furthermore, both long term exposure of HFD and HFHS were associated with increased BBB permeability (Cao et al., 2019) in the hippocampus, PFC, and striatal cortex, which was also associated with learning impairment (Kanoski et al., 2010; Davidson et al., 2012; Hargrave et al., 2016). Interestingly, some studies have shown that SCFAs modulate tight junction protein expression not just in the gut, but also in the BBB, and may therewith be associated with BBB integrity. In germ free mice, fecal transfer from pathogen-free control mice was shown to upregulate the tight junction protein occludin expression in the frontal cortex and striatum, and claudin-5 and ZO-1 expression in the hippocampus and striatum, thereby decreasing BBB permeability (Braniste et al., 2014). Moreover, in germ free mice whose intestines were mono-colonized with a single bacteria strain producing either butyrate or acetate and propionate, a normalized BBB permeability was shown, and germ-free mice who received sodium butyrate showed increased occludin expression in the frontal cortex and hippocampus (Braniste et al., 2014). It is known that the BBB endothelium also expresses monocarboxylate SCFA receptors (Vijay and Morris, 2014). Furthermore, studies in rodents showed that supplementation with sodium butyrate or monobutyrin increased the expression of occludin and ZO-1 in the brain, in contexts of HFD (Nguyen et al., 2020) or in contexts of brain damage (Li et al., 2016; Sun et al., 2016). This indicates that the BBB may be vulnerable to changes in the gut microbiota (Kelly et al., 2015), which may be modulated by SCFAs (see Figure 2).

## Discussion

On a structural level, the brains of people experiencing obesity show thinner cortices and lower brain volumes, particularly in the hippocampus and hypothalamus, as well as decreased WM integrity compared to lean adults (Puig et al., 2015; van Bloemendaal et al., 2016; Shaw et al., 2017; Hung et al., 2020). On a cognitive level, this is reflected in lower memory, verbal fluency, and executive functions (Kesse-Guyot et al., 2015; Kaur et al., 2016; Hartanto et al., 2019; Olivo et al., 2019). Recent evidence of both preclinical and clinical studies has shown multiple mechanisms underlying cognitive impairment in obesity. DIO in rodents shifted the microbiome composition, particularly increasing *Firmicutes* abundance and decreasing *Bacteroidetes* abundance (Lam et al., 2012), and led to an increased gut permeability (Hamilton et al., 2015; Schroeder et al., 2020). Furthermore, animal studies (in diet- and transgenic obese diabetic (db/db) mice and in DIO mice) have shown the effect of obesity on increased inflammation in the gut, circulation, and neuroinflammation (Duparc et al., 2011; Zhang et al., 2019), which is further linked to reduced cognitive performance (Saiyasit et al., 2020b). This neuroinflammation is primarily found in the amygdala, hippocampus and hypothalamus, being areas of emotion regulation, learning and memory, and energy metabolism, respectively (Duparc et al., 2011; Buckman et al., 2013; Almeida-Suhett et al., 2017); but also in WM (Puig et al., 2015; Samara et al., 2020). Moreover, obesity is associated with impaired cerebrovascular and BBB function, particularly once more in the hippocampus and hypothalamus (Hargrave et al., 2016; Cao et al., 2019). All together this indicates the vulnerability of brain regions such as the hippocampus and hypothalamus in obesity.

The multiple underlying processes as described above do not act alone on cognition but are closely interconnected. Both excess WAT and altered gut microbiota in obesity add to systemic inflammation (Illan-Gomez et al., 2012; Lam et al., 2012), as well as increased BBB permeability (Cao et al., 2019), leading to increased neuroinflammation (Stranahan et al., 2016) and subsequent neurodegeneration, WMH, and impaired cognition.

Interesting points for future research may include differences between race and ethnicity and sex in the role of obesity on cognitive function (Lainez et al., 2018; Metzler-Baddeley et al., 2019; Daly et al., 2020). Alzheimer’s disease is for example more prevalent in women above the age of 65 years compared to men (Hanamsagar and Bilbo, 2016). However, many rodent studies use only male animals as a homogenic group, whereas many clinical samples include more women than men. One of the well-known sex differences in obesity include differences in fat distribution, as men typically store more fat in the abdomen and women more in gluteofemoral WAT (White and Tchoukalova, 2014). Especially VAT is associated with dysregulated adipokine production and consequently inflammation and vascular disease, while gluteofemoral obesity is often associated with lower risk of metabolic disorders, however, the literature is inconsistent on this point (Kiliaan et al., 2014). These sex differences in fat distribution and therefore inflammatory state, might contribute to the sex differences seen in dementia risk factors (Azad et al., 2007; Hanamsagar and Bilbo, 2016). Furthermore, there is an interaction between sex and vascular risk factors in association with cognitive outcomes (Gannon et al., 2019). For example, in postmenopausal women hypertension and diabetes are more likely to increase to the risk of developing cognitive impairment compared to men and pre-menopausal women (Gannon et al., 2019). More research is needed to investigate the interaction between sex, differences in risk factors and cognitive outcomes. This will help to unravel underlying mechanisms and ultimately the development of personalized tailor-made treatments and preventatives such as a healthy diet and exercise.

The obesity indices used have received more attention over the last years, as it has been found that WC and WHR are more sensitive obesity indices for associations with cognitive outcomes compared to BMI. As mentioned before, VAT and SCAT were found to contain different levels of inflammation markers (McLaughlin et al., 2014), which may explain why various obesity indices are differently associated with metabolic disorders and cognitive impairments. For future research it might be interesting to discriminate between VAT and SCAT via for example the use of MRI to quantify the different WAT compartments in humans. Furthermore, including various obesity indices, as well as multiple metabolic and inflammation measures are recommended to find the underlying mechanisms causing these different outcomes in cognition. Nowadays, novel imaging techniques can measure neuroinflammation and BBB function in humans providing more information about the underlying link between obesity and cognition (Albrecht et al., 2016).

Furthermore, much evidence about the link between obesity and cognitive function in humans is based on observational cross-sectional studies which excludes any information about causality. More research is needed to study cognitive changes in obesity over time, including more information on the role of gut microbiota, inflammation and cerebrovascular function. Additionally, more research on obesity treatment is needed to investigate whether weight loss or treatment of comorbidities leads to improvement of cognitive function. Overall, obesity is associated with lower cognitive performance in the following domains: executive function, memory, inhibition, and language (Kesse-Guyot et al., 2015; Kaur et al., 2016; Hartanto et al., 2019; Olivo et al., 2019). Underlying mechanisms may include changes in gut microbiome composition which are associated with increased gut permeability and inflammation. Moreover, excess WAT and especially VAT produces pro-inflammatory adipokines, leading to low chronic systemic inflammation and reduced cerebral vascular function leading to increased BBB permeability and neuroinflammation, which may lead to neurological damage and impaired cognition (Arnoldussen et al., 2014; Hargrave et al., 2016; Cao et al., 2019). Future research is needed to investigate these pathways longitudinally, including various obesity indices and an equal gender distribution to study for example deviations in the associations between obesity measures and cognition. Moreover, early preventive measures against obesity, such as lifestyle interventions targeting healthy diet and physical activity are highly recommended to reduce the detrimental effects of obesity on brain function and structure.

## Author Contributions

All authors listed have made a substantial, direct, and intellectual contribution to the work, and approved it for publication.

## Conflict of Interest

The authors declare that the research was conducted in the absence of any commercial or financial relationships that could be construed as a potential conflict of interest.

## Publisher’s Note

All claims expressed in this article are solely those of the authors and do not necessarily represent those of their affiliated organizations, or those of the publisher, the editors and the reviewers. Any product that may be evaluated in this article, or claim that may be made by its manufacturer, is not guaranteed or endorsed by the publisher.

## Abbreviations

BBB: blood-brain barrier
BMI: body mass index
BRID: brain resource international database
CBF: cerebral blood flow
CRP: C-reactive protein
CVR: cerebrovascular reactivity
DIO: diet-induced obesity
FA: fractional anisotropy
FFA: free fatty acids
GLUT-1: glucose transporter 1
GM: gray matter
HFD: high fat diet
HFHS: high fat: high sugar
IL-1β: interleukin 1β
IL-6: interleukin 6
LABS: longitudinal assessment of bariatric surgery
LPS: lipopolysaccharide
MCP-1: monocyte chemoattractant protein-1
PAI-1: plasminogen activator inhibitor-1
SAA: serum amyloid A
SCAT: subcutaneous adipose tissue
SCFAs: short-chain fatty acids
TLR4: Toll-like receptor 4
TNF-α: tumor necrosis factor α
VAT: visceral adipose tissue
WAT: white adipose tissue
WC: waist circumference
WHR: waist-to-hip ratio
WM: white matter
WMH: white matter hyperintensities
ZO-1: zonulin-1.
