# Supplementary material for: Gut Microbiome, Inflammation, and Cerebrovascular Function: Link Between Obesity and Cognition
Source: Front Neurosci. 2021 Dec 6;15:761456. doi: 10.3389/fnins.2021.761456 (PMC8685335; doi:10.3389/fnins.2021.761456)
Supplement: Supplementary file 1 [file Table_1.pdf]

Supplementary table 1: Summary of human and rodent studies on gut microbiome and obesity.

Study	Population/Sample	Cognitive tests	Results	Additional information (e.g. age; obesity indices; observation time/FU)
Rodent studies				
Arnoldussen et al., 2017	Male LDL-/- mice, HFD or HFD with butyrate.	Morris water maze	HFD with butyrate in mid adult mice: ↓ body weight; ↓ adiposity ↓ CBF; ↓ functional connectivity; ↓ neuroinflammation.	Mice 3 mo or 6 mo old at start, dietary treatment for 3 or 6 mo.
Ashrafian et al., 2019	Male C57BL/6 mice, HFD or normal diet with *Akkermansia municiphila* or its EVs.	None	*Akkermansia municiphila* and EVs in HFD mice: ↓ body weight gain; ↓ gut permeability; ↓ gut inflammation.	Chow or HFD for 12 wk, afterwards treatment for 5 wk.
Battson et al., 2019	ob/ob and C57Bl/6j control mice, purified maintenance diet ad libitum.	None	Microbiota transplants: no effect on *Firmicutes/Bacteriodetes* ratio<br>Obese microbiota transplants to control: ↓ *Akkermansia*; ↑ *Bacteroidetes*; ↑ arterial stiffness.<br>Control microbiota transplants to obese: ↑ SCFA levels.	Mice 10 wk old at start diet, 8 wk of diet before transplantation.
Bruce-Keller et al., 2015	C57BL/6 mice, donor mice normal chow diet or HFD, recipients mice chow diet.	Elevated plus maze, open field, marble burying	Mice given HFD microbiota: ↓ time in centre/open field; ↑ marble burying; ↓ learned freezing; ↓ *Akkermansia*; ↑ *Bilophila;* ↓ occludin in jejunum; ↑ occludin in colon; ↑intestinal inflammation and permeability;<br>↑ Iba-1 and TLR4 in macrophages; ↓ ZO-1 and claudin-5 expression.	Recipient mice 3mo old, donor mice 8 wk old start diet, 10 wk of diet before transplantation.
Cheng et al., 2018	Db/db mice and C57BL/6J mice.	None	Db/db mice: ↓ tight junction ultrastructure, ↓ ZO-1 and occludin expression; ↓ NLRC3 mRNA and protein.<br>Db/db mice + butyrate: ↑ NRLC3 expression and tight junction ultrastructure.<br>NRLC3 cell lines: ↑ ZO-1 and occludin expression.<br>NRLC3 silencing in cell lines: ↓ ZO-1 and occludin expression.<br>NRLC3 overexpression + LPS: ↓ ZO-1 and occluding.<br>Caco-2 and NMC-460 cell lines + butyrate: ↑ ZO-1, occludin and NLRC3.	8 wk old mice at start, 3 wk of sodium butyrate.
De la Serre et al., 2010	Male Sprague-Dawley rats, LFD or HFD.	None	12 week HFD: ↑ inflammation and neutrophil infiltration; ↑ TLR4 immunoreactivity in ileal mucosa; ↑ plasma LPS in DIO-prone rats vs DIO-R and LFD	8 or 12 wk of diet.
Duparc et al., 2011	C57BL6/J WT and db/db male mice.	None	Db/db mice vs control: ↑ basal NO frequency; no increased NO release in hypothalamus after enteric glucose sensor stimulation →	13 to 15 wk old mice.

			disturbed gut brain axis: ↑ iNOS; ↑ IL-1β mRNA in intestines; ↑ ER stress markers; ↑ IL-1β; ↑ TNF-α in hypothalamus.	
Everard et al., 2013	Male C57BL/6 mice, chow or HFD with viable *Akkermansia* or heat-killed *Akkermansia.*	None	HFD + *Akkermansia* vs HFD: ↓ metabolic endotoxemia; ↓ adiposity; ↓ CD11c; ↓ body weight and fat mass %; ↓ hyperglycemia.<br>Heat-killed *Akkermansia* did not have this effect.	10 wk old mice at start, 4 wk of treatment.
Hamilton et al., 2015	Male Wistar rats, chow or HFD.	None	HFD vs chow: ↑ permeability in small and large intestines; ↓ IL-10 expression after 1 wk; ↑ IL1-β expression after 6 wk; ↑ ZO-1 after 3 wk; ↓ microbiota diversity.	9-10 wk old rats at start, 1,3 or 6 wk of HFD.
Lam et al., 2012	C57BL/6 female mice, chow or HFD.	None	HFD vs control: ↑ amyloid A3; ↓ adiponectin; no difference in IL-1β, IL-10, MCP-1, TNF-α, IL-6, PAI-1; ↓ ZO-1 in gut; no difference in occludin in gut; ↑ TNF-α expression; no difference in IL-6 expression; ↑ *Firmicutes*; ↓ *Bacteroidetes*; ↑ *Firmicutes/Bacteroidetes* ratio; ↓ *Lactobacillus*; ↑ *Oscillibacter* → associated with ↓ ZO-1 expression; → associated with weight gain; ↑ macrophage infiltration; ↑ TNF-α and IL-6 expression and ↑ adipocyte size in mesenteric fat.	16 wk old at start, diet for 8-12 wk.
Lam et al., 2015	C57BL/6 female mice, chow or HFD with saturated fats, HFD with n-3 PUFAs, or HFD with n-6 PUFAs.	None	HFD + saturated fats and HFD + n-6 PUFAs: ↑ body weight; ↑ fat mass.<br>HFD + saturated fats: ↑ gut permeability.<br>HFD + n-3 PUFAs: ↓ gut permeability.<br>HFD + saturated fats: ↑ fat tissue infiltration of neutrophils; ↑ CD11 mono and macrophages; ↑ total macrophages.	6 wk old mice at start, diet for 8 wk.
Lin et al., 2012	C57BL/6 male mice and Ffar3-/- male mice, chow or HFD.	None	HFD + butyrate or HFD + propionate: blocked weight gain<br>HFD + acetate: ↑ body weight<br>HFD + butyrate: ↓ food intake	3 mo old mice; diet for 4 wk.
Matt et al., 2018	Balb/c male adult and aged mice. Part 1: control mice or injection with LPS or sodium butyrate.<br>Part 2: low vs high fiber diet.	None	LPS vs control: ↑ IL-1β expression.<br>Sodium butyrate vs control: ↓ IL-1β expression.<br>LPS vs control: ↑ TNF-α expression in microglia of aged + adult mice; ↑ IL-6 expression in microglia of aged mice; ↑ IL-1β expression in hippocampus of aged + adult mice.<br>Sodium butyrate + LPS vs LPS: ↓ IL-1β expression.<br>LPS vs control: ↑ TNF-α and IL-6 expression in adult and aged mice.<br>Aged vs adult mice: difference in microbiota composition; ↓ *Mucuspirillum*; ↓ *Odoribacter*; ↑ *Ruminococcus*; ↑ *Coprococcus*; ↑ *Rikenellaceae.*	3-6 mo old mice or 22-25 mo old mice, diet for 4 wk.

			High fiber diet vs low fiber: ↓ *Ruminococcus*; ↓ *Rikenellaceae;* ↑ cecal acetate; ↑ butyrate; ↑ total SCFAs; ↓ inflammatory infiltration in gut; ↓ IL-1β, IL-1RN, IL-6, NLRP3, TLR-4 and TNF-α expression in periphery/microglia.<br>IL-1β, TNF-α and IL-6 expression in microglia were inversely associated with cecal butyrate, acetate and total SCFA levels.	
Ou et al., 2020	APP/PS1 and WT mice, chow or HFD.	Open field and Y-maze	HFD + *Akkermansia* vs HFD alone: ↑ intestinal barrier function; no decrease in Aβ in brain; no differences in cognition	3 mo old mice, diet for 6 wk.
Schroeder et al., 2020	ob/ob, WT and heterozygous wt/ob C57BL/6J mice, female NOD mice, chow diet.	None	ob/ob mice vs lean WT: ↑ body weight; ↑ insulin resistance; no differences in gut penetrability in jejunum and ileum; ↑ gut penetrability in colon, no differences in SCFAs.<br>Ob/ob mice cohoused with lean mice (where some microbiota may be carried over) repaired some mucus thickness and microbiota. This was not seen in non-obese diabetic mice.	3-4 mo old mice.
Xu et al., 2018	Db/db and C57BL/6J male mice, chow and HFD.<br>3 groups: control, with sodium butyrate or metformin.<br>Cell-culture model of colon treated with LPS and butyrate or metformin.	None	Effect butyrate in db/db mice: ↓ HbA1c; ↓ inflammatory cytokines; ↓ LPS; ↓ inflammatory cell infiltration; ↑ gut integrity; ↑ intercellular adhesion molecules; ↓ *Firmicutes/Bacteroidetes* ratio.<br>Effect of butyrate in a cell-culture model: ↑ cell proliferation; ↓ inflammatory cytokines' secretion; ↑ cell anti-oxidative stress ability; preserved epithelial monocellular integrity (damaged by LPS).	7 wk old mice at start; 5 weeks of treatment
Zhang et al., 2019	C57BL/6J male mice, standard chow or HFD with palmitic acid, divided in DIO and DR mice.	Y maze and novel object recognition	After HFD DIO showed: ↑ *Firmicutes;* ↑ *Antionobacteria*; ↓ *Bacteroides*; ↓ *Proteobacteria*; ↓ tight junction proteins; ↑ LPS; ↑ inflammation in colon and liver; ↓ recognition and spatial memory.<br>DIO vs DR mice: ↓ hippocampal BDNF.<br>↓ memory associated with ↓ *Bacteroidetes*.	6 wk old mice at start; diet for 22 wk.
Human studies				
Kong et al., 2014	55 obese and overweight subjects and 17 healthy women, divided into cluster according to diet (cluster 1 highest consumption of carbohydrates, sugar, lowest consumption of fruits, yogurt and water;	None	Differences between 3 clusters of diet (cluster 1 considered least healthy, 3 healthiest): no difference in body weight or adiposity or total energy intake.<br>Obese subjects in cluster 3 (more fruits and vegetables) vs lean subjects: ↓ circulating MCP-1; similar IL-6 levels.<br>Cluster 3 vs cluster 1+2: ↓ sCD14; no difference in other inflammatory markers; shift towards M2 macrophages; highest microbiota diversity and gene richness.	Aged 25-65 yr; BMI, fat mass, WC, cross-sectional.

	cluster 3 the other way around; cluster 2 in between cluster 1 and 3).		Within cluster 3: positive association between diet and CD163 in AT macrophages; inverse association between diet and total fat mass, adipocyte size, LDL and sCD14.<br>Obese vs lean women: ↓ *Clostridia/Bacteroidetes.*	
Tabat et al., 2020	Colon biopsies of 16 healthy adults, some biopsies treated with sodium butyrate.	None	Stimulating biopsies with stressor (C48/80) vs no stressor: ↑ colon permeability. Pre-treatment with butyrate did not affect this.<br>Stimulating biopsies with stressor + sodium butyrate vs no sodium butyrate: ↓ claudin-1.	Aged 18-65 yr; colon biopsies.
Verdam et al., 2013	28 obese and healthy weight subjects.	None	Microbiome composition clusters in 2: obese and nonobese subjects.<br>Obese vs nonobese cluster: ↓ bacterial diversity; ↓ *Bacteroidetes/Firmicutes* ratio; ↑ Proteobacteria; no intestinal permeability difference; fecal calprotectin only detectable in obese cluster; ↑ plasma CRP.<br>Plasma CRP showed positive correlation with *Bacteroidetes/Firmicutes* ratio.	Aged 19-54 yr; BMI; cross-sectional.
Combined studies				
Plovier et al., 2017	C57BL/6J male mice, chow, HFD or live/pasteurized *A. Muciniphila*.<br>Subjects with overweight/obesity and metabolic syndrome, placebo or live/pasteurized *A. muciniphila.*	None	HFD + live *Akkermansia* vs HFD: ↓ body weight; ↓ fat mass.<br>Pasteurized *Akkermansia* vs live *Akkermansia*: stronger effects.<br>HFD + *Akkermansia* vs HFD: ↓ LPS in gut.<br>Obese and overweight humans + *Akkermansia*: no effect on inflammation.	10-11 wk old mice, treatment for 4 or 5 wk.<br>20 humans treatment for 3mo.

FU: follow-up; LDL: low-density lipoprotein; HFD: high fat diet; CBF: cerebral blood flow; mo: month(s); EVs: extracellular vesicles; wk: week(s); SCFA: short-chain fatty acid; Iba-1: ionized calcium binding adaptor molecule-1; TLR4: Toll-like receptor 4; ZO-1: zonula occludens-1; NLRC3: nucleotide-binding oligomerization domain (NOD)-like receptor family pyrin domain-containing 3; LFD: low fat diet; LPS: lipopolysaccharide; DIO: diet-induced obesity; WT: wild-type; NO: nitric oxide; iNOS: inducible nitric oxide synthase; IL-1β: interleukin 1β; ER: endoplasmic reticulum; TNF-α: tumor necrosis factor α; IL-10: interleukin 10; MCP-1: monocyte chemotactic protein-1; PAI: plasminogen activator inhibitor; n-6 PUFAs: omega-6 polyunsaturated fatty acids; n-3 PUFAs: omega-3 polyunsaturated fatty acids; IL-1RN: interleukin 1 receptor antagonist gene; NLRP3: NLR family pyrin domain containing 3; Aβ: amyloid beta; HbA1c: hemoglobin A1c; BDNF: brain-derived neurotrophic factor; DR: diet resistant; AT: adipose tissue; yr: year(s); WC: waist circumference; CRP: c-reactive protein; BMI: body mass index.
