# Supplementary material for: Gut Microbiome, Inflammation, and Cerebrovascular Function: Link Between Obesity and Cognition
Source: Front Neurosci. 2021 Dec 6;15:761456. doi: 10.3389/fnins.2021.761456 (PMC8685335; doi:10.3389/fnins.2021.761456)
Supplement: Supplementary file 2 [file Table_2.pdf]

Supplementary table 2: Summary of human and rodent studies on gut microbiome and cognition in obesity.

Study	Population/Sample	Cognitive tests	Results	Additional information (e.g. age; obesity indices; observation time/FU)
Rodent studies				
Bruce-Keller et al., 2015	C57BL/6 mice, donor mice normal chow diet or HFD, recipients mice chow diet.	Elevated plus maze, open field, marble burying	Mice given HFD microbiota: ↓ time in centre/open field; ↑ marble burying; ↓ learned freezing; ↓ *Akkermansia*; ↑ *Bilophila;* ↓ occludin in jejunum; ↑ occludin in colon; ↑intestinal inflammation and permeability; ↑ Iba-1 and TLR4 in macrophages; ↓ ZO-1 and claudin-5 expression.	Recipient mice 3 mo old, donor mice 8 wk old start diet, 10 wk of diet before transplantation.
Frohlich et al., 2016	C57BL/6N male mice, standard chow with AB or vehicle.	Open field test; elevated plus maze; novel object recognition test; Barnes maze	AB treatment: ↓ bacterial load; ↓ diversity; ↓ novel object recognition memory; no effect learning & memory; no effect on claudin5, TJP1 and occludin expression in medial PFC and hypothalamus; ↓ claudin 5 and occludin expression in hippocampus.<br>Molecules in the brain related to learning and memory were also affected by AB.	8-11 wk old mice; treatment for 11 days.
Gareau et al., 2011	SPF C57BL/6 female mice, GF Swiss-Webster female mice and age-matched SPF Swiss-Webster control female mice; infected with *C rodentium* or sham, and exposed to stress (water avoidance stress).	Novel object test; T-maze, light/dark box	Infection alone did not affect behaviour in C57BL/6 mice. Infection + stress: ↓ memory.<br>Memory dysfunction prevented by probiotics in infected mice.<br>Memory impairment in GF mice with or without stress vs control mice.	5-6 wk old mice at start; testing 10 or 30 days after infection.
Saiyasit et al., 2020b	Male Wistar rats, chow or HFD.	Open field, Morris water maze	HFD after 2 wk: gut dysbiosis.<br>HFD after 8 wk: insulin resistance.<br>HFD after 12 wk: hippocampal synaptic dysplasticity; ↓ dendritic spine density; ↑ ionized calcium-binding adapter molecule 1^+ cells; ↑ hippocampal ROS and apoptosis with cognitive decline.<br>HFD after 20 & 40 weeks: ↓ % resident microglia; ↑ % infiltrated macrophages.<br>HFD after 40 wk: ↑ Aβ brain levels.	Treatment for 2, 8, 12, 20 or 40 wk.
Zhang et al., 2019	C57BL/6J male mice, standard chow or HFD with	Y maze and novel object recognition	After HFD DIO showed: ↑ *Firmicutes;* ↑ *Antionobacteria*; ↓ *Bacterioides*; ↓ *Proteobacteria*; ↓ tight junction proteins; ↑ LPS; ↑ inflammation in colon and liver; ↓ recognition and spatial memory.	6 wk old mice at start; diet for 22 wk.

	palmitic acid, divided in DIO and DR mice.		DIO vs DR mice: ↓ hippocampal BDNF. ↓ memory associated with ↓ *Bacteroidetes*.	
Human studies				
Arnoriaga-Rodriguez et al., 2021	Multiple cohorts with subjects with and without obesity.	Stroop, Iowa Gambling, Wisconsin Card Sorting	Gut microbiome linked to several inhibitory control tests and associated brain structures. Human donors with impairment in inhibitory control led to recipient mice with impairment in inhibitory control.	Multiple age ranges; BMI, WC, fat mass; longitudinal and cross-sectional.
Kreutzer et al., 2017	57 obese and 54 matched nonobese subjects.	Hypothalamic inflammation via MRI.	Obese subjects vs non-obese: T2 hyperintensities in left mediobasal hypothalamus, which was positively associated with systemic inflammation. Inverse association between mediobasal hypothalamus inflammation and *Parasutterella sp.* and *Marinilabiliaceae.* The abundance of these microbiota is influences by nutritional intake of fat (measured by FFQ).	Aged 33-54 yr; BMI; cross-sectional.
Combined studies				
Arnoriaga-Rodriguez et al., 2020	51 subjects without obesity, 65 subjects with obesity. Male C57BL/6J mice on chow. Microbiota transplantation from human donors to mice.	Verbal learning and memory; digit span	Positive association between *Firmicutes* and memory; inverse association between *Bacteroidetes* and *Proteobacteria* and memory. Microbiota from obese humans in mice: ↓ memory scores. Human donors with ↑ memory: recipient mice ↑ memory.	Aged 27.2-66.6 yr; cross-sectional

HFD: high fat diet; Iba-1: ionized calcium binding adaptor molecule-1; TLR4: Toll-like receptor 4; ZO-1: zonula occludens-1; mo: month(s); AB: antibiotics; TJP1: tight junction protein 1; PFC: prefrontal cortex; GF: germ-free; SPF: specific pathogen-free; ROS: reactive oxygen species; Aβ: amyloid beta; DIO: diet-induced obesity; DR: diet resistant; LPS: lipopolysaccharide; BDNF: brain-derived neurotrophic factor; BMI: body mass index; WC: waist circumference; FFQ: food frequency questionnaire; yr: year(s).
