# Supplementary material for: Gut Microbiome, Inflammation, and Cerebrovascular Function: Link Between Obesity and Cognition
Source: Front Neurosci. 2021 Dec 6;15:761456. doi: 10.3389/fnins.2021.761456 (PMC8685335; doi:10.3389/fnins.2021.761456)
Supplement: Supplementary file 3 [file Table_3.pdf]

Supplementary table 3: Summary of rodent and human studies on WAT inflammation, adipokines and obesity.

Study	Population/Sample	Results	Additional information (e.g. age; obesity indices; observation time/FU)
Human studies			
Bilgic Gazioglu et al., 2015	Obese subjects with (n=37) and without (n=20) CAD.	Obese patient with CAD vs without CAD: ↑ PAI-1; ↑ TNF-α; ↑ PAI-1 mRNA expression in mediastinal AT; ↑ TNF-α in epicardial AT, mediastinal AT and subcutaneous AT.	Average age around 58 yr; BMI, WC; cross-sectional.
Clemente-Postigo et al., 2019	33 obese subjects scheduled for surgery, divided into high and low LPS groups.	High LPS group vs low LPS group: ↑ triglycerides; ↓ expression of genes for AT function and lipogenesis; ↑ expression of inflammatory genes in visceral and subcutaneous AT.<br>*In vitro* experiments with adipocytes stimulated by LPS: ↑ pro-inflammatory cytokines.	Average age around 44 yr; BMI; cross-sectional.
Deguchi et al., 2010	113 VTE patients and 113 matched controls.	VTE patients vs controls: ↑ SAA level.<br>SAA correlated positively with CRP and fibrinogen	No information on age; cross-sectional.
Illan-Gomez et al. 2012	60 obese female BS patients and 30 healthy lean women.	12 mo after surgery: ↑ adiponectin; ↑ HDL cholesterol; ↓ IL-6; ↓ hs-CRP; ↓ cholesterol; ↓ triglycerides; ↓ LDL cholesterol; ↓ glucose; ↓ insulin; ↓ HOMA.<br>At 12 mo positive correlations between IL-6 and BMI, insulin and HOMA.<br>At 12 mo positive correlations between hs-CRP and BMI, triglycerides, insulin and HOMA.	Average age around 40 yr; BMI, WHR; over-time.
McLaughlin et al., 2014	20 BS patients and 47 healthy subjects.	VAT and SAT from BS patients show CD4 and CD8 T cell infiltration.<br>VAT vs SAT: ↑ Th-1; ↑ Th17; ↑ CD8 T cells.<br>T-cell profiles in VAT correlated with SAT and peripheral blood.<br>Th2 correlated inversely with hs-CRP and system insulin resistance.<br>Th1 in SAT correlating with IL-6.<br>IL-1- expression in AT was inversely associated with insulin resistance.	Aged 35-65 yr; cross-sectional.

sWD: western diet; wk; week(s) IL-10: interleukin 10; VAT: visceral adipose tissue; yr: year(s); BMI: body mass index; WC: waist circumference; LPS: lipopolysaccharide; VTE: venous thromboembolism; SAA: serum amyloid a; BS: bariatric surgery; CRP: c-reactive protein; mo: month(s); HDL: high-density lipoprotein; IL-6: interleukin 6; hs-CRP: high-sensitivity C-reactive protein; LDL: low-density lipoprotein; HOMA: homeostasis model assessment; WHR: waist-hip ratio; SAT: subcutaneous adipose tissue.
