# Supplementary material for: Gut Microbiome, Inflammation, and Cerebrovascular Function: Link Between Obesity and Cognition
Source: Front Neurosci. 2021 Dec 6;15:761456. doi: 10.3389/fnins.2021.761456 (PMC8685335; doi:10.3389/fnins.2021.761456)
Supplement: Supplementary file 4 [file Table_4.pdf]

Supplementary table 4: Summary of studies on obesity related neuroinflammation.

Study	Population/Sample	Cognitive tests and neuroimaging	Results	Additional information (e.g. age; obesity indices; observation time/FU)
Rodent studies				
Almeida-Suhett et al., 2017	C57BL/6J male mice, chow or HFD.	Open field; elevated zero maze; Y maze, forced swim test	After 16 wk of HFD: cognitive impairment on Y-maze test; ↑ anxiety during open field and elevated zero maze test; ↑ depressive behaviour in forced swim test; ↑ IL-1β expression in the hippocampus and amygdala; ↑ hippocampal GFAP and Iba-1.<br>Cognitive performance was negatively correlated with IL-1β in hippocampus and amygdala.<br>Anxiety-like behaviour was positively correlated with IL-1β in the amygdala.	5 wk old mice at start; treatment of 18 wk.
Buckman et al., 2013	C57BL/6, MC4R deficient, or Tie2-GFP female mice, chow or HFD.	None	Genetically and HFD- induced obese mice vs lean mice: ↑ GFAP immunoreactivity in several nuclei of hypothalamus. Distinct pattern between regions of the hypothalamus. No differences between genetic model or diet-induced obesity.<br>GFAP immunoreactivity was often associated with microvasculature.	12-17 wk old mice at start; 20 wk of diet.
Duparc et al., 2011	C57BL6/J WT and db/db male mice.	None	Db/db mice vs control: ↑ basal NO frequency; no increased NO release in hypothalamus after enteric glucose sensor stimulation → disturbed gut brain axis; ↑ iNOS, ↑ IL-1β mRNA in intestines; ↑ ER stress markers; ↑ IL-1β, ↑ TNF-α in hypothalamus.	13 to 15 wk old mice.
Erion et al., 2014	C57BL/6J WT and db/db male mice, with or without treadmill training, fat transplantation, with or without IL-1ra.	Y maze, novel object recognition	db/db without treadmill vs control and db/db with treadmill: ↓ spatial recognition; ↓ object recognition memory; infrequent dendritic spines; ↑ Iba-1 in hippocampus and epididymal fat; ↑ circulating IL-1β; ↑ MHCII immunoreactivity in hippocampus.<br>Db/db with treadmill showed LTP deficit reversion.<br>Fat transplantation from db/db to wt control: ↓ spatial and object memory; ↑ MHCII; ↑ CD163 cells; ↑ Iba-1 in hippocampus.<br>Effects lipectomy: ↑ spatial and object memory; ↑ LPS; ↓ CD163 cells; ↓ MHCII and Iba-1 in hippocampus.<br>IL-1ra infusion in hippocampus abolished memory deficits in db/db mice, but did not affect time exploring in maze, it normalized LTP and dendritic spine density compared to WT mice.	5 wk old mice at start; 10 wk of treadmill training; 2 wk of IL-1ra treatment.

Gladding et al,. 2018	C57BL/6J male mice, LFD or HFD, insulin or saline infusion.	Morris water maze and Y maze	HFD vs LFD: ↓ spatial memory; ↑ inflammatory markers in hippocampus. Both were reversed by insulin infusion in the hippocampus.	8 wk old mice at start; 12 wk of diet; 4 wk of insulin infusion treatment.
Hao et al., 2016	C57BL/6J male mice, LFD or HFD or first HFD then LFD.	Y maze, novel object preference test	HFD-induced obesity: ↓ hippocampus-dependent memory; ↓ LTP; ↑ MHCII in hippocampal microglia; ↑ IL-1β in hippocampus.<br>Diet reversal from HFD to LFD: reduced adiposity partially; MHCII normalized; plasticity deficits normalized.<br>LFD and HFD/LFD: ↑ synaptic density near microglial processes, but not in HFD.	6 wk old mice at start; 1,2,3, 5 mo of HFD or HFD for 3mo then LFD for 2mo.
Jin et al., 2020	C57BL/6J and ob/ob male mice.	None	Ob/ob mice vs WT mice: ↑ body weight; ↑ fat mass; insulin sensitivity; ↑ pro-inflammatory cytokines in serum and hippocampus; ↓ brain weight; ↓ hippocampal volume; ↓ Sorl1 and BDNF in hippocampus; ↑ Wdfy1 gene; ↑ LCN2 and MMP9; levels in serum and hippocampus.	5 wk old mice at start; measurement at 25 wk.
Lainez et al., 2018	C57BL/6J male and female mice, chow or HFD.	None	Male and ovariectomized females + HFD vs female + HFD: ↑ weight gain (protection by ovarian hormones).<br>Males vs females: ↑ weight gonadal fat, no differences in other depots.<br>Obese male mice show microglial activation and macrophage infiltration in hypothalamus, females are protected for these inflammatory changes.<br>Obese female mice vs obese male mice: ↑ IL-10 in hypothalamus.<br>Obese male mice vs obese female mice: ↓ PSD95 in hypothalamus.	4 wk old at start; 12 wk of diet.
Lee et al., 2018	LysMGFP and LysMtdT male mice, chow or HFD.	None	HFD consumption: GFP in hypothalamic ARC; change in morphology from linear to ramifying; expansion of GFP cells; ↑ iNOS, ↑ Hif-1α, ↑ IL-1β, ↑ IL-6 and ↑ TNF-α mRNA in MBH; ↓ Vegfa, ↓ eNOS mRNA.	7 wk old at start; 4 or 20 wk of diet.
Park et al., 2019	C57/BL6 male mice, chow or HFD.<br>Ob/ob mice on chow.	None	HFD vs chow: different gene expression pattern in hypothalamus; ↑ inflammatory response; ↑ IFNy; ↑ adipogenesis; ↑ TNF-α signalling.<br>Ob/ob and HFD- induced obese mice: ↑ TNF-α signalling and enriched gene sets of cancer pathways.	4 wk old mice at start; 20 wk of diet.<br>9 wk old ob/ob mice.
Saiyasit et al., 2020a	Male Wistar rats, chow or HFD.	None	HFD after 12 and 40 wk: ↑ plasma NT; metabolic disturbances; ↑ systemic inflammation; ↑ oxidative stress; ↑ Iba-1 in hippocampus; ↓ dendritic spines in hippocampus<br>HFD after 40 wk: ↑ brain NT.	12 or 40 wk of diet.
Human studies				
Franz et al., 2019	Vietnam Era Twin Study of Aging. 173 men with obesity and	None	At age 62, obese vs non-obese: ↓ cortex thickness in frontal and temporal lobe areas. Frontal lobe thinness mainly in right hemisphere.<br>No differences between groups in WM volume	Measures at average age 20, 40, 56, and 62 yr; BMI; longitudinal.

	202 men without obesity.			
Kaur et al., 2016	61 adults without history of heart failures.	MMSE, IQ test, verbal learning test, long delay free recall, recognition discriminability, trail making test, COWA, digit span	Higher WHR associated with: ↓ COWA score; ↓ BDNF levels. BDNF fully mediated the relation between WHR and COWA.	Aged 40-60 yr; BMI, WHR; cross-sectional.
Klinedinst et al., 2019	4431 adults from the UK Biobank.	Fluid intelligence test	↑ lean muscle mass predicted improvement in fluid intelligence.<br>↑ visceral and non-visceral adipose mass predicted decline in fluid intelligence.<br>The effects of visceral adipose mass on fluid intelligence were mediated by leukocytes.	Aged 40-79 yr; lean muscle mass, non-visceral adipose mass, visceral adipose mass; longitudinal.
Kullman et al., 2020	115 normal weight, overweight and obese adults without T2D.	MRI	Global brain water content (indirect measure of inflammation) not associated with any obesity indices.<br>Regional: ↑ BMI associated with: ↑ water content in cerebellum, limbic lobe and sub-lobular region.<br>VAT strongest tested link between water content and obesity.	Aged 24-74 yr; BMI, WC, WHR, body fat distribution; cross-sectional.
Lauridsen et al., 2017	141 Post-mortem samples from BrainCloud database, without neurological or psychiatric diseases.	None	↑ BMI associated with: ↓ IL-10; ↑ iNOS; not with IL-1β, IL-6 or PTGS2.<br>Expression of IL-10 most affected by BMI ≥ 40.	Aged 18-78 yr; BMI; post-mortem samples.
Puig et al., 2015	24 obese and 24 healthy women.	Wechsler adult intelligence scale, trail making test, Wisconsin card sorting, Stroop, Iowa gambling task; DTI	Obese subjects showed ↓ λ1 values in hypothalamus.<br>Subject with higher λ1 values: ↑ BMI; ↑ fat mass; ↑ inflammatory markers; ↑ carotid-intima media thickness; ↑ hepatic steatosis; ↓ scores on cognitive tests.	Average age 49 yr; BMI, WC, fat mass; cross-sectional
Samara et al., 2020	2 cohort, in total: 43 obese and 62 non-obese participants.	Extensive cognitive battery:	Obese vs lean: ↑ DBSI-RF; ↓ DBSI-FF in multiple WM tracts.<br>Regional analyses, obese vs lean: ↑ DBSI-RF and ↓ DBSI-FF in hippocampus.	Average age around 30 yr; BMI; cross-sectional

		executive function, verbal and visuospatial learning and memory and motor speed; DBSI; DTI	Hippocampal and amygdalar DBSI-FF and DBSI-FF associated with cognitive performance in cohort 2.	
Shaw et al., 2017	404 midlife individuals.	MRI	Obesity associated with ↓ cortical thickness in right frontal cortex.<br>↑ BMI over 8 yr is associated with cortical thinning in the posterior cingulate, right lingual gyrus, anterior cingulate, and peri-calcarine sulcus.	Aged 44-49 yr at baseline; BMI; longitudinal.
Combined studies				
Baufeld et al., 2016	C57BL6J male mice, chow or HFD.<br>Brain autopsies of 9 non-obese and 12 obese cases.	None	8 wk of HFD: ↑ microglia number; ↑ GFAP astrocytes in hypothalamus. This reaction was limited to endogenous microglia and not mediated by infiltrating myeloid cells.<br>3 days of HFD: ↑ gene expression of IL-1β and IL-6.<br>8 wk of HFD: ↓ expression of IL-6 in human cases with obesity showed hypothalamic gliosis and more microglia dystrophy. The ratio of hypothalamic/cortical Iba-1 area was larger in obese subjects and correlated with BMI.	Mice: 14-17 wk old mice at start; diet for 3 days to 20 wk.<br>Human: average age around 67 yr. Cross-sectional.
Thaler et al., 2012	Male Long-Evans rats, male C56BL/6 mice on chow or HFD and 34 human participants with MRI scan.	MRI	1 to 3 days of HFD in mice and rats: ↑ IL-6, TNF-α, Socs3, IkBkB mRNA in hypothalamus.<br>8 days of HFD in mice and rats: ↑ GFAP in arcuate nucleus, returning to baseline after few wk.<br>8 wk HFD: ↑ microglia in arcuate nucleus.<br>20 wk of HFD in mice and rats: ↑ autophagosomes mainly in POMC. Mitochondria in HFD in POMC were irregular, swelled, less electron dense lumen.<br>In humans, T2 left MBH/amygdala signal ratio intensity was positively correlated with BMI, but not with age or sex and was higher in obese subjects compared to lean subjects, but BMI was not correlated with putamen/amygdala signal ratio intensity.	Rats and mice: 1 day to 8 mo of diet.<br>Humans: aged 18-63 yr; BMI; retrospective cohort study.

HFD: high fat diet; IL-1β: interleukin 1β; GFAP: glial fibrillary acidic protein; Iba-1: ionized calcium binding adaptor molecule-1; wk: week(s); IL-6: interleukin 6; NO: nitric oxide; iNOS: inducible nitric oxide synthase; IL-1β: interleukin 1β; ER: endoplasmic reticulum; TNF-α: tumor necrosis factor α; MHCII: major histocompatibility complex class II; LTP: long-term potentiation; LPS: lipopolysaccharide; IL-1ra: interleukin 1 receptor antagonist; WT: wild-type; LFD: low fat diet; mo: month(s); Sorl1: sortilin-related receptor 1; BDNF: brain-derived neurotrophic factor; Wdfy1: WD repeat and FYVE-domain-containing 1; LCN2: lipocalin 2; MMP9: matrix metalloproteinase 9; IL-10: interleukin 10; PSD95: postsynaptic density protein 95; GFP: green fluorescent

protein; tdT: tdTomato; ARC: arcuate nucleus; Hif-1α: hypoxia-inducible factor-1α; MBH: mediobasal hypothalamus; Vegfa: vascular endothelial growth factor-A; eNOS: endothelial NOS; IFNγ: interferon gamma; NT: neurotensin; WM: white matter; BMI: body mass index; yr: year(s); MMSE: mini-mental state examination; IQ: intelligence quotient; COWA: controlled oral word association; WHR: waist-hip ratio; T2D: type 2 diabetes; VAT: visceral adipose tissue; WC: waist circumference; DTI: diffusion tensor imaging; λ1: eigenvalue 1; DBSI: diffusion basis spectrum imaging; DBSI-RF: DBSI-derived restricted fraction; DBSI-FF: DBSI-derived fiber fraction; MRI: magnetic resonance imaging; Socs3: suppressor of cytokine signaling 3; IkBkB: inhibitor of nuclear factor kappa B kinase subunit beta; POMC: proopiomelanocortin.
